# The Economic Burden of Mental Disorders in the European Union: A Cross-Country Assessment

**DOI:** 10.1192/j.eurpsy.2026.11644

**Published:** 2026-07-31

**Authors:** F. Tatsis, S. Georgakis, E. Dragioti, M. Gouva

**Affiliations:** 1 Department of Medicine; 2 Scientific Laboratory of Psychology and Person-Centered Care, Department of Nursing, University of Ioannina, Ioannina, Greece

## Abstract

**Introduction:**

Mental disorders constitute a growing public health and economic challenge across the European Union. Conditions such as depression, anxiety, and severe psychiatric illnesses are increasingly prevalent, especially among working-age populations. While their clinical and societal impact is well documented, their economic consequences are often underestimated in health policy planning. Understanding the scale and composition of this burden is essential for effective resource allocation and strategic health system design.

**Objectives:**

This study aims to assess the total economic burden of mental health disorders across EU member states using existing international data. Specifically, it seeks to quantify both direct and indirect costs, evaluate cross-country differences, and highlight the policy implications for national mental health strategies.

**Methods:**

This investigation is based on data sourced from international institutions including the OECD, WHO Europe, and Eurostat, as well as published studies between 2015 and 2025. A narrative synthesis approach was employed to consolidate data on the financial impact of mental health disorders in EU countries. The economic burden was disaggregated into direct costs (hospitalization, pharmaceuticals, outpatient care) and indirect costs (productivity loss, early retirement, and disability). Data were analyzed comparatively to reflect both absolute expenditures and burden as a percentage of national GDP.

**Results:**

The analysis shows considerable variation in the economic burden of mental disorders across the EU. Germany bears the highest cost, at approximately €125 billion annually, followed by France (€100B), Italy (€90B), and Spain (€75B), in line with prior European estimates. In all countries, indirect costs (such as productivity loss, early retirement and disability) exceed direct healthcare costs, accounting for 60% to 70% of the total burden. In Greece, for example, indirect costs reach 70%, reflecting broader EU patterns. As a share of GDP, the burden ranges from 3.5% to 4.5%, with Germany at the upper end, underscoring the economic significance of mental health.

**Conclusions:**

Mental health disorders represent a major economic burden for EU member states, with indirect costs consistently surpassing direct healthcare spending. Despite their scale, mental health expenditures remain underprioritized in many national health budgets. These findings support the urgent need for expanded investment in prevention, early intervention, and rehabilitation services. From an economic perspective, improving mental health is not only a public health priority but a strategic investment in productivity and social stability across the European Union.

**Disclosure of Interest:**

None Declared

